# Transcriptome analysis unravels key pathways and hub genes related to immature fruit abscission in *Camellia oleifera*

**DOI:** 10.3389/fpls.2024.1418358

**Published:** 2024-08-09

**Authors:** Xiaoling Ma, Shiwen Chen, Jinwen Li, Xiang Ouyang

**Affiliations:** ^1^ Key Laboratory of Cultivation and Protection for Non-Wood Forest Trees of the Ministry of Education, Central South University of Forestry and Technology, Changsha, China; ^2^ State Key Laboratory of Hybrid Rice, Hunan Hybrid Rice Research Center (HHRRC), Changsha, China

**Keywords:** *Camellia oleifera*, fruit abscission, phytohormones, transcriptome, hub genes

## Abstract

Immature fruit abscission of *Camellia oleifera* (*C. oleifera*) is a common problem limiting yield increases. However, the regulatory mechanisms underlying immature fruit abscission in *C. oleifera* are unclear. In this study, we systematically investigated changes in the morphological, physiological, and gene expression of fruit abscission zones (FAZs) of soon-to-abscise fruits (M2). We found that fruit abscission before ripening mainly occurs during the August abscission stage of ‘Huashuo’. At the beginning of this stage, the FAZs of M2 have a marked dent, and the separation layer structures are preliminarily formed. Phytohormone analysis showed that the contents of indole-3-acetic acid (IAA) and jasmonic acid (JA) in the FAZs of M2 were significantly decreased compared with the non-abscised fruits, while the content of *trans*-zeatin (TZR) was increased. Transcriptome analysis identified differentially expressed genes (DEGs) mainly involved in phytohormone metabolism, including ethylene, auxin, JA, and the *cis*-zeatin signal transduction pathway. There were also many DEGs involved in cell wall catabolism. Weighted gene co-expression network analysis (WGCNA) further suggested that the transcription factors *NAC100* and *ERF114* participate in the immature fruit abscission of *C. oleifera*. This study provides insights into the fruit abscission mechanism of *C. oleifera*.

## 1 Introduction

*Camellia oleifera* (*C. oleifera*) is a perennial evergreen shrub or small tree of the *Camellia* genus, Theaceae (Ji et al., 2022). *C. oleifera* is widely distributed in the hilly areas of southern China and is one of the world’s four major woody oil trees, alongside oil palm, olive, and coconut (Zhang et al., 2021). *C. oleifera* oil (tea oil) extracted from seeds is commonly known as ‘eastern olive oil’ with high nutritional and health value. Tea oil contains more than 90% unsaturated fatty acids, such as oleic acid and linoleic acid, which can soften blood vessels, lower blood lipids, and blood pressure (Pan et al., 2012; Lin et al., 2022b). In addition, *C. oleifera* meal and shells can be processed into chemicals and industrial raw materials to manufacture soap, green fertilizer, activated carbon, and so forth (Zhang et al., 2021; Li et al., 2023a). With the rapid development of the *C. oleifera* industry, improving the yield of *C. oleifera* is particularly important. However, immature fruit abscission is the main reason restricting *C. oleifera* yield production increase (Hu et al., 2021).

Abscission is the process by which plant organs separate from the parent plant; it is a ubiquitous phenomenon throughout the plant life cycle (Patharkar and Walker, 2018). Organ abscission requires forming a unique structure called the abscission zone (AZ), which constitutes the area where the organ is separated from the plant body (Kim et al., 2019). Organ abscission can be summarized into four basic phases: (i) differentiation of the AZ; (ii) acquisition of competence of the AZ cells to respond to abscission signals; (iii) activation of the AZ cells, cell wall modification, and cell separation; and (iv) differentiation of a protective layer on the plant separation surface (Tucker and Kim, 2015; Kim et al., 2019).

The occurrence of organ abscission is influenced by developmental and environmental factors (Taylor and Whitelaw, 2001; Estornell et al., 2013). Furthermore, phytohormones were also demonstrated to play important roles in organ abscission, which is based on a complex signaling system of hormone synthesis, catabolism, and transport (Sawicki et al., 2015; Chen et al., 2023). Ethylene (ETH) and auxin are considered to be the central regulators that affect organ abscission (Meir et al., 2015; Zhao et al., 2020). The ethylene-insensitive mutants, *etr1-1* and *ein2* exhibit delayed abscission of floral organs in *Arabidopsis thaliana* (Patterson and Bleecker, 2004). The key genes of ethylene synthesis, *1-aminocyclopropane-1-carboxylic acid oxidases* (*CoACOs*) were confirmed as highly positively related to fruit abscission in *C. oleifera* (Hu et al., 2021). The *arf1* and *arf2* mutant plants exhibited delays in the abscission of floral organs (Ellis et al., 2005). *PpILR1*, which encodes an indole-3-acetic acid (IAA)-amino hydrolase, and overexpressing *PpILR1* exhibited premature pedicel abscission (Wang et al., 2021). In addition, abscisic acid (ABA), cytokinin (CTX), jasmonic acid (JA), gibberellin (GA), and brassinosteroid (BR) have been demonstrated to affect organ abscission (Sawicki et al., 2015; Kim et al., 2019; Li et al., 2023b).

Abscission is achieved by the dissolution of the cell wall within the AZ. The primary cell wall is mainly composed of cellulose, hemicellulose (cross-linking glycans), pectin, lignin, and structural proteins. Modifications in the genes which regulate cell wall structural components have been confirmed to affect fruit abscission (Estornell et al., 2013; Kim et al., 2019). For example, it was reported that overexpression of the litchi *LcPG2* can promote the floral organ abscission of Arabidopsis (Ma et al., 2020). Two cellulases (*LcCEL2* and *LcCEL8*) strongly associated with abscission can be directly up-regulated by the LcHB2 transcription factor in litchi (Li et al., 2019). *BEL1-LIKE HOMEODOMAIN 4* (*SlBL4*) is involved in chloroplast development and cell wall metabolism in tomato, while silencing *SlBL4* resulted in the enlargement and pre-abscission of the tomato (Yan et al., 2021). However, the regulating mechanisms of fruit abscission in *C. oleifera* remain largely unknown.

Previously, we showed that ethylene was associated with fruit abscission of *C. oleifera* (Hu et al., 2021). In this study, we analyzed the dynamics of fruit abscission during fruit development of *C. oleifera* ‘Huashuo’ by comparing the changes in the structure and endogenous hormone content of fruit abscission zones (FAZs) between non-abscised fruit and soon-to-abscised fruit during the peak fruit abscission period. Key genes affecting *C. oleifera* fruit abscission were identified through transcriptome sequencing analysis of FAZs from non-abscised fruits, abnormal fruit, and fruit treated with ethephon.

## 2 Materials and methods

### 2.1 Plant materials and treatments

The trees of *C. oleifera* cultivar ‘Huashuo’ (9-year-old, high-crown grafting) were used in this study. The oil-tea tree experimental station (113° 21’ E, 28° 05’ N) of Central South University of Forestry and Technology was in Dongcheng Town, Wangcheng District, Hunan Province, China. Nine trees with similar growth potential were selected randomly to calculate the fruit abscission rate in ‘Huashuo’ on 15 May, 15 June, 15 July, 15 August, 30 August (August abscission stage), 15 September, and 15 October 2019. The fruit abscission rate was calculated referencing our previous study (Hu et al., 2021). The different developmental periods FAZs of ‘Huashuo’ were collected by cutting 1 mm at each side of the abscission fracture plane and stored at -80°C to measure the phytohormones content from May 2019 to October 2019. In this study, ‘Huashuo’ trees were treated on 22 August 2019 with water (control, CK) and 2 g L^-1^ ethephon solution (ETH) respectively, according to our previous paper (Hu et al., 2021). FAZs of CK and ETH were gathered and designated as CK1 and ETH1 on the 8^th^ day after treatments. Due to the fact that the 16^th^ day sampling period after treatments was set to the August abscission stage, FAZs of the non-abscised and soon-to-abscise fruits obtained from CK were named CK2 and M2, respectively, while the FAZs of ETH treated on the 16^th^ day were named ETH2. Three biological replicates were arranged for each treatment in this study, each containing 8 FAZs, and the FAZs collected by cutting 1 mm at each side of the abscission fracture plane from three trees were termed as one biological replicate. The FAZs of each sample (CK2, M2, and ETH2) were collected for cytological observation. The FAZs of CK1, ETH1, CK2, M2, and ETH2 were immediately soaked in liquid nitrogen and stored in the -80°C freezer for subsequent RNA extraction and determination of phytohormone content.

### 2.2 Morphological and cytological observation of FAZs in *C. oleifera*

To observe the morphological differences in the FAZs between the non-abscised fruits and soon-to-abscised fruits, FAZs samples of CK2 and M2 were collected on the same day for camera photography analysis and observed using a light microscope. In addition, the samples collected on the same day were fixed in FAA’s solution (35%-40% methanal: acetic acid: 70% ethanol=5:5:90, v/v/v) about 24 h, firstly softened in hydrogen peroxide-acetic acid (1:1, v/v) for 6 h and secondly softened in 70% tert-butyl alcohol-glycerol mixture (1:1, v/v) about 4 days, then dehydrated in the tert-butyl alcohol series (50, 70, 85, 95 and 100%), each dehydrated steps for 30 min and repeated these steps. After infiltration into safranine and fast green, followed by decoloration using dimethylbenzene, specimens were embedded in paraffin according to the conventional paraffin sectioning method (Fan et al., 2017). Samples were sectioned into 10 μm sections by a Leica RM2265 microtome (Leica Camera AG, Solms, Germany), and the sections were observed using a microscope (BX51, Olympus, Tokyo Japan).

### 2.3 Determination of endogenous hormone content in *C. oleifera* FAZs

The phytohormones, including indole-3-acetic acid (IAA), indole-3-butyric acid (IBA), trans-zeatin (TZR), *cis*-zeatin, isoamyl alkenyl adenine (IP), isopentenyl adenosine (IPA), ABA, GA1/3/4/7, JA, and salicylic acid (SA), at the different abscission statuses (CK2 and M2) FAZs were determined to quantify the different hormones. The quantification of these hormones was determined according to previous studies (Lin et al., 2022a). Each eluted fraction was evaporated, reconstituted with 200 μL methanol, and subjected to a high-performance liquid chromatography-tandem mass spectrometry (HPLC-MS/MS) system (6500, Agilent, Santa Clara, CA, USA). To further clarify the key hormones that affect fruit abscission, we quantified IAA, JA, TZR, and ethylene in FAZs during the developmental period from May 2019 to October 2019. The 1-aminocyclopropane-1-carboxylic acid (ACC) is the ethylene precursor. ACC content and 1-aminocyclopropane-1-carboxylic acid oxidase (ACO) activity, reflecting ethylene content, were measured according to our previous study (Hu et al., 2021). All physiological indexes were determined in triplicate biological replicates.

### 2.4 RNA preparation, transcriptome sequencing, and assembly

A total of 15 samples (CK1_1, CK1_2, CK1_3, ETH1_1, ETH1_2, ETH1_3, CK2_1, CK2_2, CK2_3, ETH2_1, ETH2_2, ETH2_3, M2_1, M2_2, M2_3) were subjected to RNA-seq analysis in this study. Arabic numerals after underscored digital indicate three biological replicates of the corresponding samples. The total RNA of each sample was isolated using Trizol reagent (Invitrogen, USA) according to the manufacturer’s instructions (Li et al., 2023a). RNA degradation and contamination were analyzed using agarose gels. The purity and integrity of the samples were determined using a NanoPhotometer spectrophotometer (IMPLEN, Germany) and an RNA Nano 6000 Assay Kit with an Agilent 2100 bioanalyzer system (Aligent, USA), respectively. The construction of the cDNA library and Illumina sequencing were performed by the Gene Denovo Biotechnology Co. (Guangzhou, China). After removing the adaptor sequences and low-quality reads, high-quality reads from all samples were assembled using Trinity to construct unique consensus sequences for reference (Li et al., 2023a). Gene functions were annotated according to the databases as follows: Non-redundant (Nr) protein database (Pruitt et al., 2007), SwissProt database (Bairoch and Boeckmann, 1992), Clusters of Orthologous Groups of proteins (COG) database (Tatusov et al., 2003), and Kyoto Encyclopedia of Genes and Genomes (KEGG) protein pathway database (Kanehisa et al., 2008).

### 2.5 Differential expression analysis and weighted gene co-expression network analysis

Gene expression levels were calculated and normalized to fragments per kilobase of transcript per million mapped reads (FPKM) by expectation maximization (RSEM) for each sample. Differential expression analysis was performed using DESeq2 (Love et al., 2014). The differentially expressed genes (DEGs) in ETH-treated and M2 samples were compared with CK samples using the DESeq software with a false discovery rate < 0.05 and absolute fold change ≥2. The differentially expressed gene sets were obtained by analyzing the differential expression between two sample groups, and the gene sets were named CK1 vs ETH1 (CK1-ETH1), CK2 vs ETH2 (CK2-ETH2), CK2 vs M2 (CK2-M2), and ETH2 vs M2 (ETH2-M2) in the analysis results.

We constructed co-expression networks using the WGCNA (v1.47) package in R (Langfelder and Horvath, 2009) to identify the hub genes involved in fruit abscission. After filtering, 23839 genes were used to construct co-expression modules using the automatic network construction function and block-wise module detection with a soft threshold of eight. Parameters 50 minModulesize and 0.3 mergeCutHeight were selected to merge similar transcripts. The eigengene value of each module was calculated and used to examine its association with each trait. Visualization of the gene co-expression network was conducted using the Cytoscape platform (Shannon et al., 2003).

### 2.6 qRT-PCR analysis

The gene-specific primers for qRT-PCR were designed using Primer Premier 5 software and synthesized by Tsingke Biotechnology (Beijing, China). The primers are listed in **Supplementary Table S1**. The first-strand cDNA was synthesized using the Fast Quant RT Kit (Tiangen, Beijing, China) according to the manufacturer’s instructions. qRT-PCR analysis was performed with a LightCycler 480 (Roche, Basel, Switzerland) system using the SYBR Green I Master Kit (Roche, Basel, Switzerland). The relative expression levels were calculated with the formula of 2^-ΔΔCt^ (Livak and Schmittgen, 2001). *CoEF1α* (elongation factor) was adopted as the internal control (Hu et al., 2021).

### 2.7 Statistical analysis

All data were collected in triplicate, and the results are presented as means ± SD. Microsoft Office Excel 2013 was used to process the data. The statistical analysis of the means was performed using one-way ANOVA with the SPSS 20.0 package for Windows (IBM, New York, NY, USA). Ducan’s multiple range tests with *P* < 0.05 indicate significant differences.

## 3 Results

### 3.1 *C. oleifera* ‘Huashuo’ exhibits obvious fruit abscission during the August abscission stage

**Figure 1:**
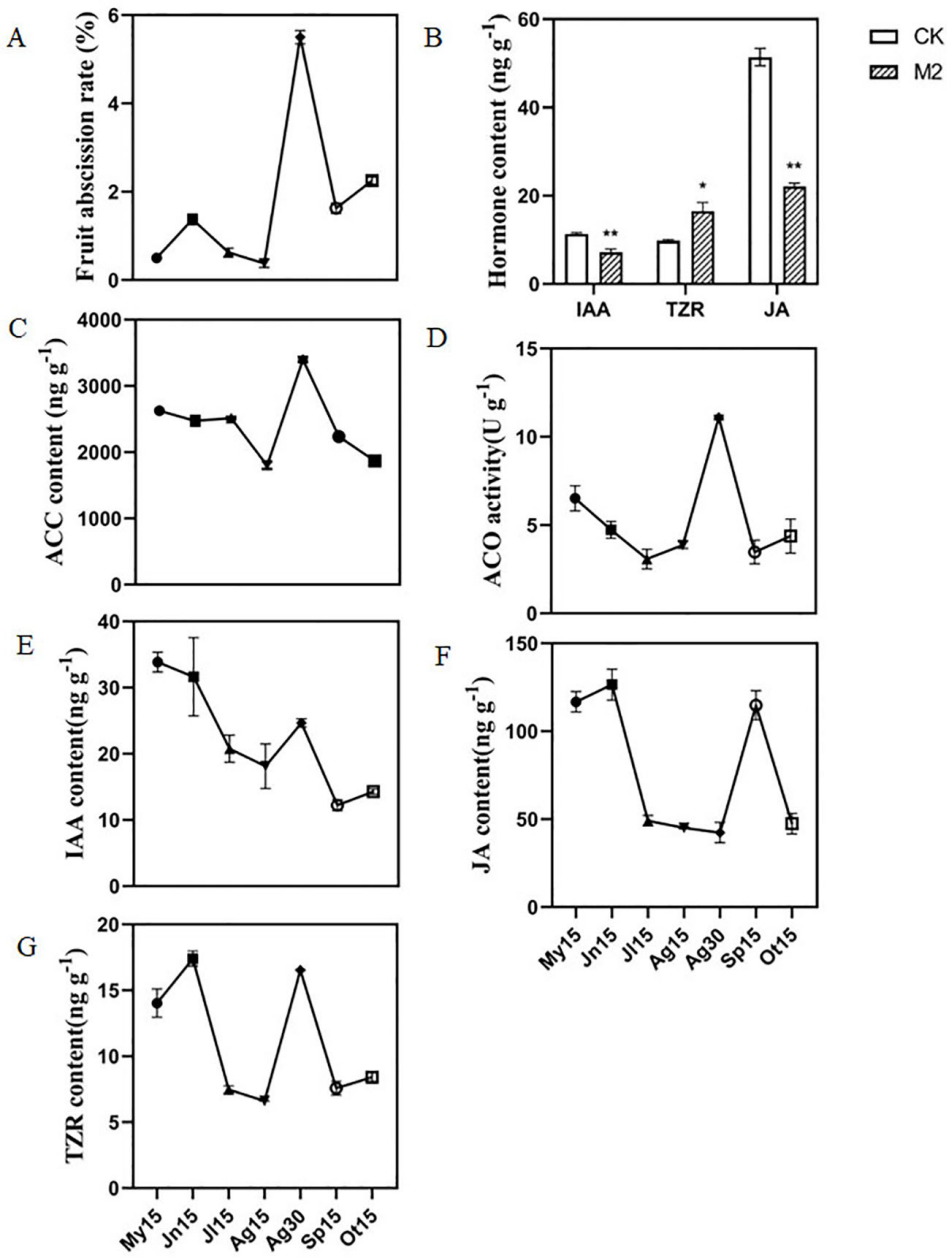
Changes in fruit abscission rate and hormone content in the FAZs of *C. oleifera* ‘Huashuo’. **(A)** Fruit abscission rate of *C*. *oleifera* at different stages of fruit development; **(B)** The IAA, TZR, and JA contents in the FAZs of CK2 and M2; **(C)** ACC content, **(D)** ACO activity, **(E)** IAA content, **(F)** JA content, and **(G)** TZR content in the FAZs of *C*. *oleifera* at different stages of fruit development. My15, Jn15, Jl15, Ag15, Ag30, Sp15, and Ot15 represent May 15, June 15, July 15, August 15, August 30, September 15, and October 15, respectively; FAZs, fruit abscission zones; IAA, indole-3-acetic acid; TZR, trans-zeatin; JA, jasmonic acid; CK2 and M2 represent the FAZs of the non-abscised and soon-to-abscised fruits, respectively. Single and double asterisks indicate differences at *P* < 0.05 and *P* < 0.01, respectively.

To determine the dynamics of fruit abscission during fruit development in *C. oleifera*, the fruit abscission rate of ‘Huashuo’ was investigated from May 2019 to October 2019. The fruit abscission rate had a relatively low basal level during fruit development from May to October 2019 (except for August 30). Moreover, the fruit abscission rate showed a peak level on August 30 and then decreased on September 15 and October 15 (**Figure 1A**). These results showed that *C. oleifera* ‘Huashuo’ had a significant fruit abscission on August 30, which had a significant impact on the yield of *C. oleifera*. Therefore, our subsequent research will mainly focus on the fruit abscission during this period.

### 3.2 Morphological and cytological characteristics of FAZs were changed in soon-to-abscise fruits

**Figure 2:**
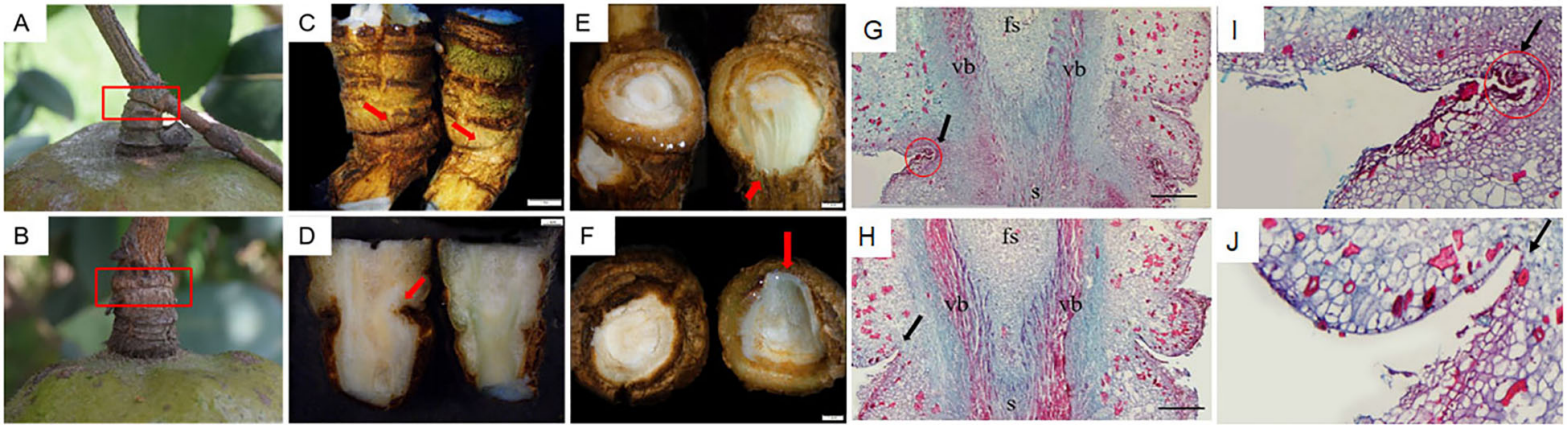
Morphological and cytological observation of FAZs in abscised fruits (M2) and non-abscised fruits (CK2). **(A)** FAZs of soon-to-abscised fruits; **(B)** FAZs of non-abscised fruits; **(C)** FAZs of soon-to-abscised fruits (left) and non-abscised fruits (right) under a stereomicroscope; **(D)** Longitudinal section of the FAZs in soon-to-abscised fruits (left) and non-abscised fruits (right) under a stereomicroscope; **(E)** The proximal end of the FAZs between the soon-to-abscised fruits (left) and non-abscised fruits (right); **(F)** The distal end of the FAZs between the soon-to-abscised fruits (left) and non-abscised fruits (right); **(G, I)** Cytological observation of FAZs in soon-to-abscised fruits; **(H, J)** Cytological observation of FAZs in non-abscised fruits. The red boxes in **(A, B)** show the FAZs of soon-to-abscised fruits and non-abscised fruits, respectively; The red arrows in **(C, D)** show the junctions of fruit stalk and stem; The red arrows in **(E, F)** show the cross sections at the proximal and distal ends of FAZs in non-abscised fruits; The black arrows in **(G, I)** show the layer cells morphology in the FAZs of soon-to-abscised fruits, and the black arrows in **(H, J)** show the cell morphology in the FAZs of non-abscised fruits; The red circles in **(G, I)** show the lysed cells of FAZs in soon-to-abscised fruits; fs, fruit stalk; s, stem; vb, vascular bundle. Bars indicate 1.0 mm in the fruit image.

Previously, we found that the FAZs of *C. oleifera* are located at the junction of fruit stalk and stem (Hu et al., 2021). To accurately clarify the early morphological differences of FAZs between the non-abscised fruits and soon-to-abscised fruits, we analyzed the morphological and cytological changes in the FAZs of fruits which are soon-to-abscised (M2) during the August abscission stage. In M2, we found the junction surface of the fruit stalk and stem had a marked dent, while the dent was not obvious in CK2 (**Figures 2A, B**). The longitudinal section of FAZs in CK2 presented a vibrant bright green medulla, but the inner parts of FAZs in M2 showed slightly yellow and dry (**Figures 2C, D**). Moreover, when compared with CK2, the M2 had a deep pit, and the epidermis and cortex at the proximal and distal ends were dark brown, and there were no obvious tearing marks after the fruit was removed (**Figures 2E, F**).

Paraffin sections were further made to observe the FAZ longitudinal sections of CK2 and M2. We found the cortical tissues of M2 were heavily indented compared with CK2, indicating a higher number of cells that had already been lysed (**Figures 2G, I**). Moreover, the separation layer structure has preliminarily formed in FAZs of M2. These layers of cells are closely packed, have a smaller volume than neighboring cells, and are more likely to be dyed purple-red by Muscovy (**Figures 2G, I**), indicating a higher degree of lignification than normal cells. However, the FAZ cells of CK2 were arranged orderly, and there was no significant difference between them and adjacent cells in morphology (**Figures 2H, J**). These results showed that the FAZs of M2 had a marked dent and that the internal structure distinctly changed.

### 3.3 Endogenous hormone contents were changed in developing fruit abscission zones

Our previous result showed that ethylene plays an important role in the fruit abscission of *C. oleifera* (Hu et al., 2021). To understand how phytohormones influenced fruit abscission, the contents of phytohormones were systematically determined in FAZs of CK2 and M2. The results showed that the IAA and JA contents in M2 were 7.18 and 22.05 ng g^-1^, respectively, which were significantly decreased compared with CK2 by 36.56% and 57.08%, respectively. On the contrary, the TZR content in M2 (16.54 ng g^-1^) was increased by 69.20% compared with CK2 (9.77 ng g^-1^) (**Figure 1B**). We found no differences in the contents of the other 11 endogenous hormones, including the ABA, between M2 and CK2 (**Supplementary Table S2**).

To further assess relationships between the four phytohormones (ethylene, IAA, JA, and TZR) in FAZs and the fruit abscission rate, we evaluated the dynamics of phytohormone response systems in FAZs during fruit development. The ACC content and ACO activity exhibited a highly positively related change pattern with the fruit abscission rate, with the highest level on August 30 reaching 3397.61 ng g^-1^ and 11.09 U g^-1^, respectively (**Figures 1C, D**). This is consistent with our previous reports, ACC content, and ACO activity were significantly increased in FAZs under ethephon treatment (Hu et al., 2021). Interestingly, there is an opposite change pattern of IAA content, which was found gradually decrease throughout the developmental period examined except on August 30 (**Figure 1E**). The JA contents had two high peak levels on June 15 and September 15, while the lower JA amounts were from July 15 to August 30 (**Figure 1F**). In addition, the higher TZR amounts were from May 15 to June 15 and on August 30, while the lower TZR amounts were from July 15 to August 15 and September 15 to October 15 (**Figure 1G**).

### 3.4 Global analysis of RNA sequencing data

**Figure 3:**
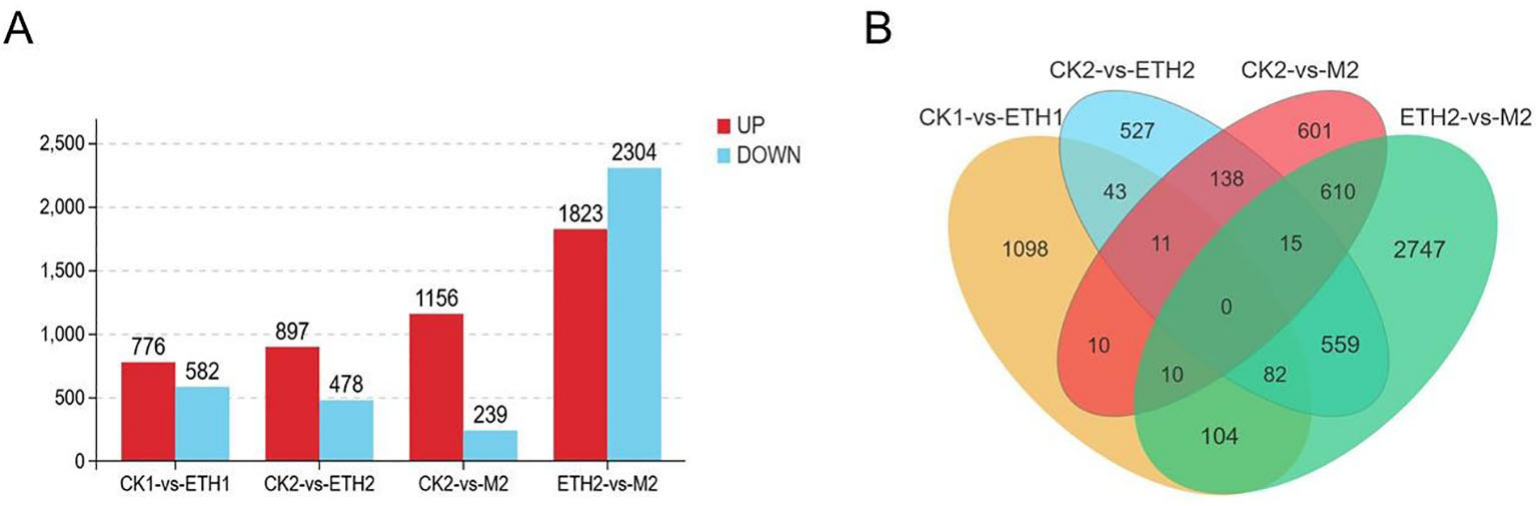
Differentially expressed genes (DEGs) of *C*. *oleifera* FAZs in different growth states. **(A)** Number of up- and down-regulated DEGs in the CK1 vs ETH1, CK2 vs ETH2, CK2 vs M2, and ETH2 vs M2 differential expression gene sets; **(B)** Venn diagram of all DEGs numbers among the four gene sets.

To identify potential regulatory mechanisms that lead to the immature fruit abscission of *C. oleifera*, the comparative transcriptome analyses of FAZs were performed. A total of 15 samples which were assigned to five groups were sequenced. Four comparison groups were conducted, including CK1-ETH1, CK2-ETH2, CK2-M2, and ETH2-M2. The numbers of DEGs in the four comparison groups were 1358, 1375, 1395, and 4127, respectively. Specifically, 776 and 582 DEGs were up and down-regulated in CK1-ETH1, 897 and 478 in CK2-ETH2, 1156 and 239 in CK2-M2, and 1823 and 2304 in ETH2-M2, respectively (**Figure 3A**). The higher upregulated ratios of DEGs in CK1-ETH1, CK2-ETH2, and CK2-M2 comparison groups and lower upregulated ratios of DEGs in ETH2-M2 suggest the ethephon treatment suppresses the expression of some genes. Venn results further showed there were 11 mutual DEGs in the CK1-ETH1, CK2-ETH2, and CK2-M2 (**Figure 3B**), indicating that these genes are good candidates to be involved in fruit abscission. For example, although the mutual DEG *ACO1* was significantly down-regulated in ETH1 compared with CK1, it was significantly up-regulated in both ETH2 and M2 compared with CK2.

GO analysis showed that the most significantly enriched GO terms were metabolic process, cell part, and catalytic activity (**Supplementary Figure S1**). KEGG analysis displayed the DEGs of CK2-M2 were significantly enriched in pentose and glucuronate interconversions, phenylpropanoid biosynthesis, and glycolysis/gluconeogenesis (**Supplementary Figure S2**). This suggests that the organ and developmentally specific pattern of metabolites were affected in M2. The KEGG results of CK1-ETH1, CK2-ETH2, and ETH2-M2 were significantly enriched in metabolic pathways or biosynthesis of secondary metabolites. These results indicated that exogenous spraying of ethephon had effects on the biological metabolic processes of FAZs, which led to fruit abscission. Interestingly, DEGs in all four comparisons were found significantly enriched in the ‘plant hormone signal transduction pathway’. This suggests that plant hormone signal transduction is strongly associated with fruit abscission in *C. oleifera* FAZs.

### 3.5 Specific DEGs involved in phytohormone biosynthesis

**Figure 4:**
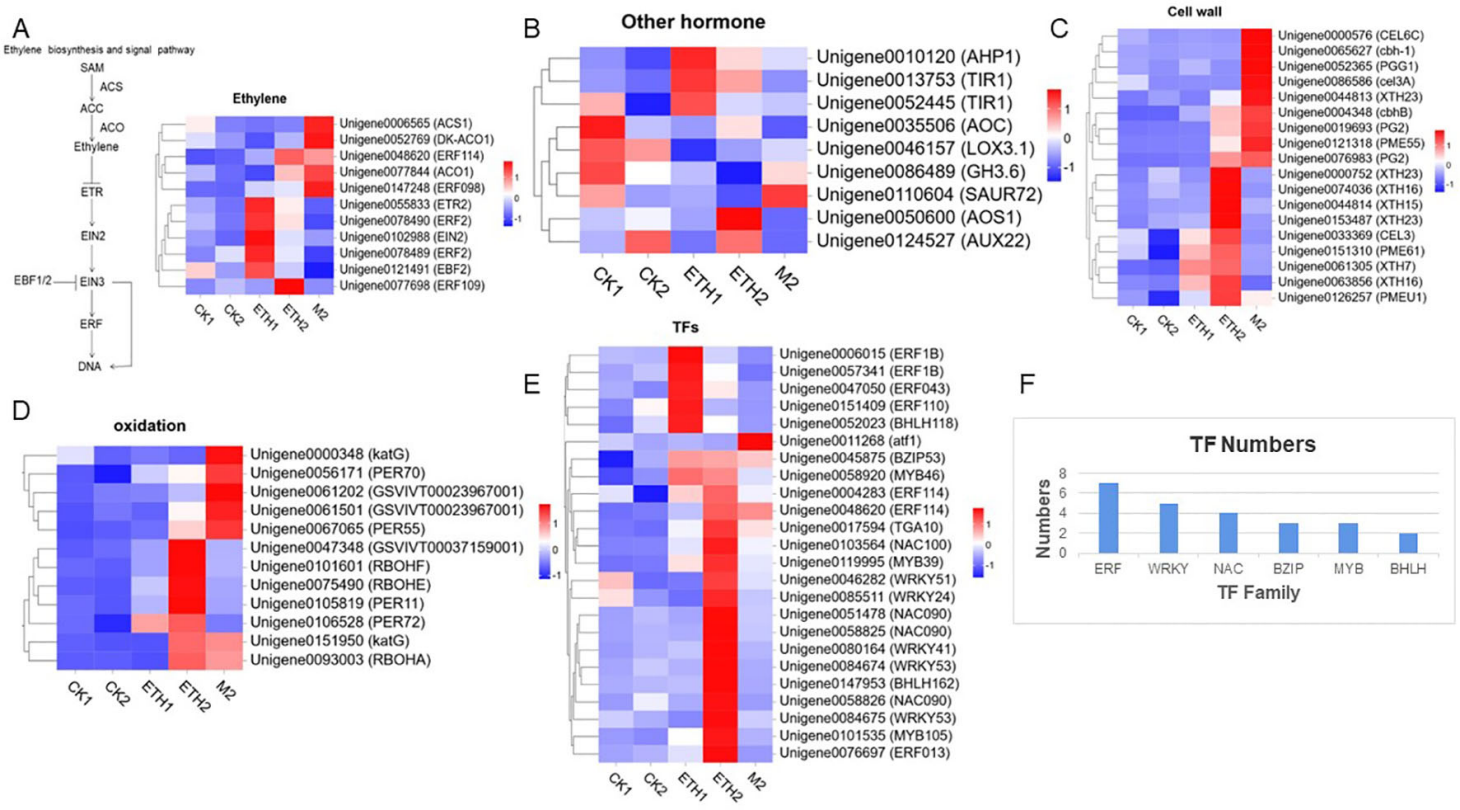
Key gene expression patterns related to the abscission of *C*. *oleifera*. **(A)** DEGs related to the ethylene signaling pathway; **(B)** DEGs related to other hormones; **(C)** DEGs related to the cell wall; **(D)** DEGs related to oxidation; **(E)** DEGs related to transcription factor; **(F)** DEGs numbers of transcription factor belonging to different gene families.

There were 11 DEGs involved in ethylene biosynthesis and signal transduction identified in CK1-ETH1, CK2-ETH2 and CK2-M2 comparison groups (**Figure 4A**). Two ethylene synthesis genes, 1-aminocyclopropane-1-carboxylic acid synthase (*ACS*) and *ACO* were found significantly up-regulated in M2 (**Figure 4A**). The genes *ETHYLENE RECEPTOR 2* (*ETR2*), *ETHYLENE INSENSITIVE PROTEIN 2* (*EIN2*), and *ERF* which involved in ethylene signal transduction pathway were up-regulated in ETH1 and ETH2. However, *EIN3 BINDING PROTEIN 2* (*EBF2*) was found down-regulated in M2 (**Figure 4A**). These suggest that ethylene plays a critical role in the fruit abscission of *C. oleifera*.

We also identified 9 DEGs related to IAA, JA, and TZR (**Figure 4B**). The *TRANSPORT INHIBITION RESPONSE 1* (*TIR1*) was significantly up-regulated, whereas the early auxin response genes (*GH3.6*, *SAUR72*, and *AUX22*) were significantly down-regulated after ethephon treatment (**Figure 4B**). The *LIPOXYGENASE 3.1* (*LOX3.1*), *ALLENE OXIDE SYNTHASE 1* (*AOS1*), and *ALLENE OXIDE CYCLASE* (*AOC*) involved in JA biosynthesis were significantly down-regulated in ETH1 and M2 when compared with CK (**Figure 4B**). In addition, *ARABIDOPSIS PHOSPHOTRANSFER PROTEIN 1* (*AHP1*) which is involved in *cis*-zeatin nucleoside signal transduction was up-regulated both in ETH1, ETH2, and M2 (**Figure 4B**). These results show that the IAA, JA, and TZR might also play important roles in *C. oleifera* fruit abscission.

### 3.6 DEGs which encode transcription factors and enzymes involved in cell wall catabolism and oxidation

To clarify the regulating mechanisms of changes in cell morphological differences of FAZs between the non-abscised fruits and soon-to-abscised fruits, 18 DEGs related to cellulose and pectin decomposition were identified (**Figure 4C**). Heat map analysis showed the cellulase (*CEL*), exoglucanase (*CEL6C*) and cellobiohydrolase (*CBH*) were highly expressed in M2. Polygalacturonase (*PG2*), which is associated with pectinase-mediated cell wall decomposition, also showed a high expression level in M2, and the expression level was up-regulated by more than 50-fold compared with that of CK2. In addition, pectinesterase (*PME*) was significantly up-regulated in ETH2 and M2 compared with CK2. The endoglycosidase (*XTH*), which is involved in hemicellulose decomposition also showed a high expression level in ETH2 (**Figure 4C**).

According to the KEGG results, many genes related to oxidation were enriched in the ‘metabolic pathway’ or ‘secondary metabolites’ pathways; therefore we speculate that the abscission of *C. oleifera* fruit may be related to the expression of oxidation genes. Twelve DEGs related to NADPH and oxidase peroxidase of the three comparisons were identified (**Figure 4D**). Two NADPH oxidase genes (*RBOHF*, *RBOHE*) were significantly up-regulated in ETH2. At the same time, most peroxidase-related genes were significantly up-regulated in ETH2 and M2 compared with CK2. It is worth noting that the expression levels of *KATG* and *PER70* at M2 were more than 10 times higher than those of CK2, with the most significant change in *KATG* expression. Interestingly, the genes NADPH oxidase (*RBOHA*) and peroxidase (*KATG*) were significantly up-regulated in both ETH2 and M2 relative to CK2 (**Figure 4D**), indicating that these two genes have a certain effect under ethephon treatment and natural abscission. These results suggest that oxidation in the FAZs of *C. oleifera* has an important effect on fruit abscission.

We identified 171 transcription factors from three comparisons of DEGs, of which 24 may be related to fruit abscission, and belong to gene families such as *ERF*, *WRKY*, *NAC*, *BZIP*, *MYB*, and *BHLH* (**Figures 4E, F**). These genes in the FAZs of ethylene-induced or natural fruit abscission were significantly up-regulated compared with the control. For example, genes including two *ERFs* (*ERF013* and *ERF1B*), two *WRKYs* (*WRKY53* and *WRKY41*), two *NACs* (*NAC100* and *NAC090*), and one *BHLH* (*BHLH162*) were significantly up-regulated after ethephon treatment. In addition, the *BZIP* gene (*ATF1*) was significantly up-regulated under natural fruit abscission, indicating that this gene may mediate fruit abscission under natural conditions. Moreover, one *ERF* (*ERF114*) and two *BZIPs* (*BZIP53* and *TGA10*) were significantly up-regulated during both ethephon and natural abscission (**Figure 4E**), indicating that these genes are highly related to fruit abscission.

### 3.7 Co-expression network analysis

**Figure 5:**
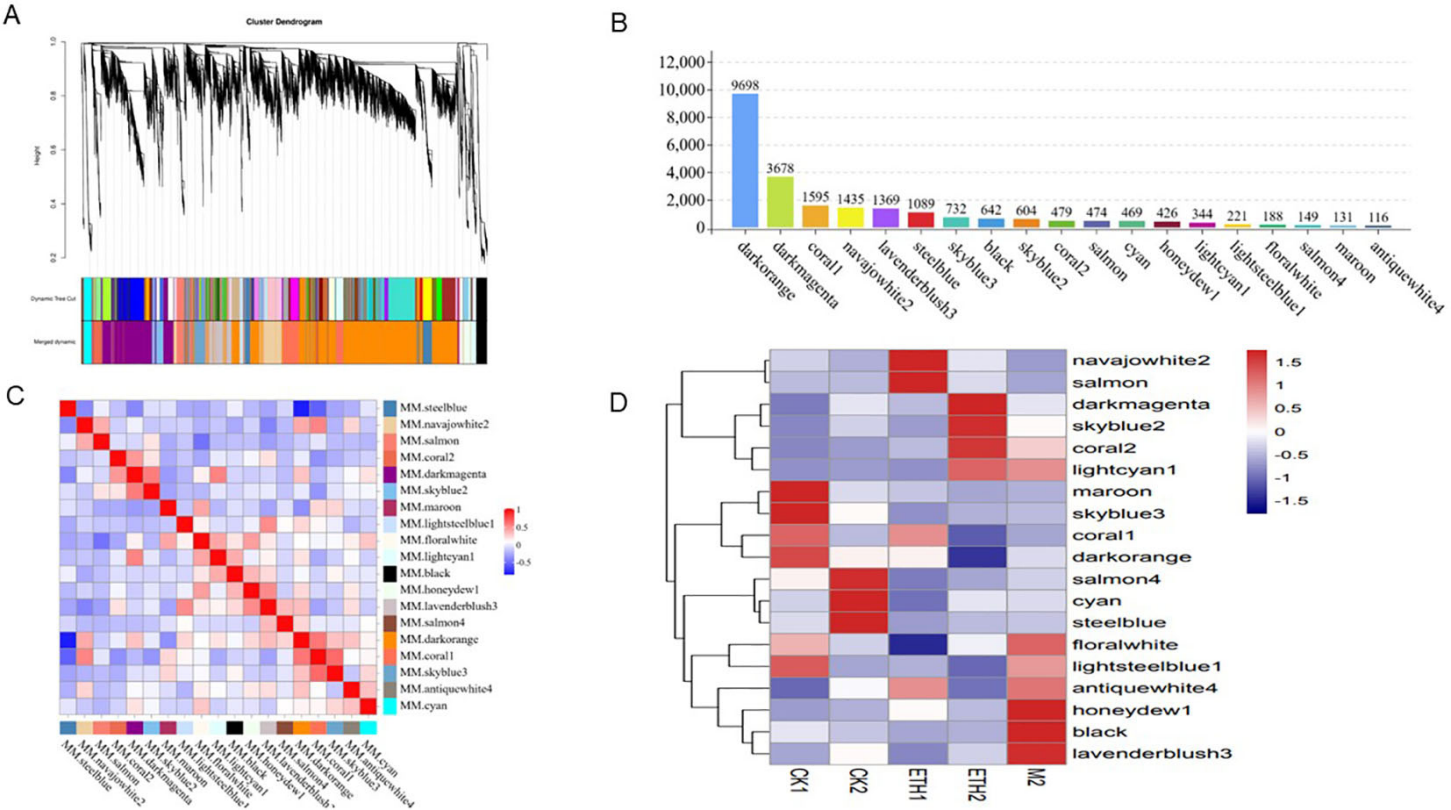
Weighted gene co-expression network analysis (WGCNA) results obtained using RNA sequencing data. **(A)** Clustering dendrograms of genes; Dissimilarity was based on topological, overlap, and assigned module colors; **(B)** Number of genes in 19 modules; **(C)** Correlations between 19 modules; **(D)** Relationships between different groups and modules.

WGCNA was conducted to further identify genes related to fruit abscission. We identified 19 co-expressed modules with gene numbers ranging from 116 (antiquewhite4) to 9698 (dark orange) (**Figures 5A, B**). Correlations between modules are shown in **Figure 5C**. We paid special attention to three modules (antiquewhite4, coral2, and lightcyan1) related to fruit abscission: the antiquewhite4 module showed high correlation at ETH1 and M2, and low correlation at CK1 and CK2; the coral2 and lightcyan1 module were highly correlated at ETH2 and M2, and lowly correlated at CK2 (**Figure 5D**). In addition, KEGG results for these three modules showed that the antiquewhite4 module was significantly enriched in peroxisome and butanoate metabolism; the coral2 module was significantly enriched in tryptophan metabolism, starch and sucrose metabolism, pentose and glucuronate interconversions, galactose metabolism, and arginine and proline metabolism. The lightcyanl module was significantly enriched in metabolic pathways, biosynthesis of secondary metabolites, and carbon metabolism (**Supplementary Figure S3**). These results showed that: Peroxisomes related to oxidation, the genes of lysine degradation, histidine metabolism, and tryptophan metabolism related to cell metabolism were enriched.

**Figure 6:**
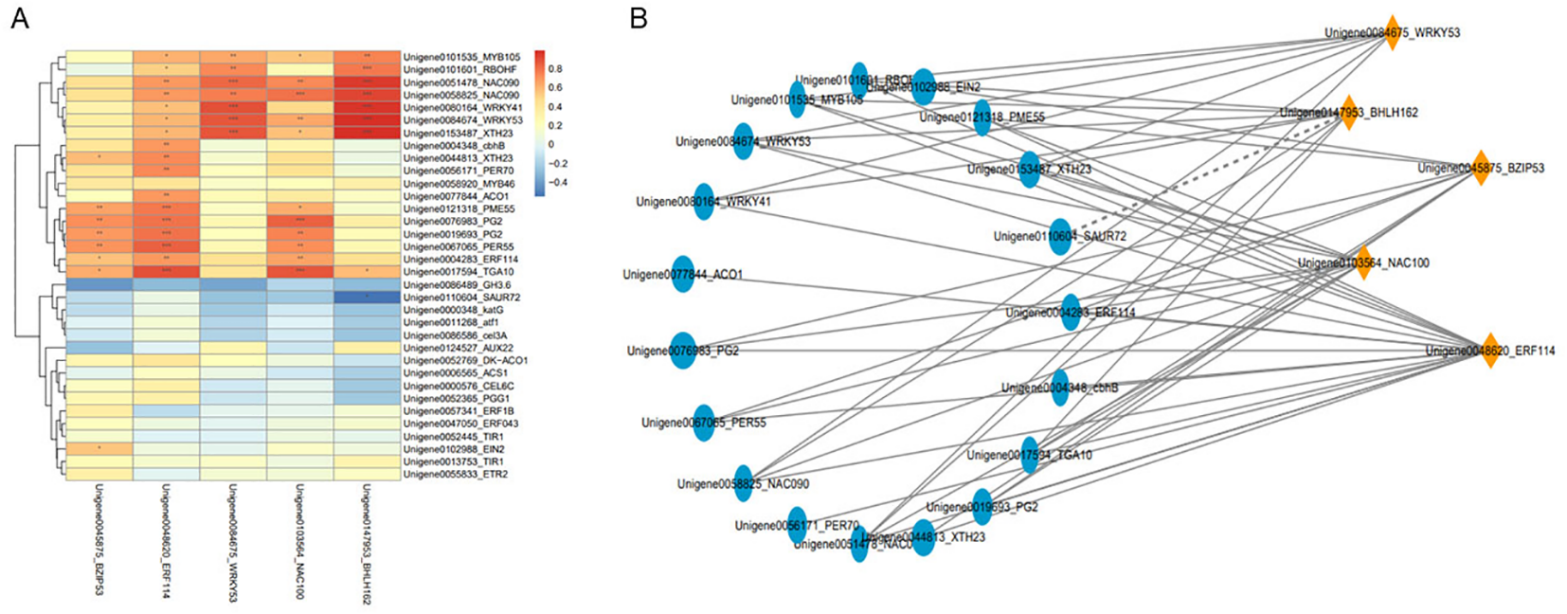
Relationship between key genes involved in fruit abscission and TFs. **(A)** Heatmap of fruit abscission-related genes and key TFs; **(B)** Correlation network diagram of fruit abscission genes and key TFs.

We conducted co-expression network analyses of 39 genes (cell wall catabolism, phytohormone biosynthesis, oxidation, and transcription factors) related to fruit abscission that were screened. The transcription factors *NAC100*, *ERF114*, *BZIP53*, *BHLH162*, and *WRKY53* were identified as hub genes (**Figure 6A**). To further identify genes significantly related to the 5 hub genes, we used the correlation between genes (*P*<0.05) as the screening criteria and plotted the correlation map between the 5 hub genes and 19 other significantly related genes (**Figure 6B**). The results show that *NAC100* and *ERF114* are the most important transcription factors affecting fruit abscission based on gene connectivity. The qRT-PCR results of *NAC100* and *ERF114* also showed higher expression under two abscission statuses (M2 and ETH2) compared with the control (**Supplementary Figure S4**). These results suggest that *NAC100* and *ERF114* are good candidates for regulating fruit abscission.

## 4 Discussion

### 4.1 Significance of phytohormones in the regulation of *C. oleifera* fruit abscission

Phytohormones are important chemical substances produced by plants that are involved in the regulation of growth and development of plants and responses to biotic and abiotic stresses (Peleg and Blumwald, 2011). Previous studies have identified ethylene and ABA as the main abscission inducers in camellia and blue honeysuckle (Cai et al., 2022; Chen et al., 2023). In this study, although we found no differences in ABA content between the FAZs of M2 and CK2 (**Supplementary Table S2**), the content of ethylene was confirmed greatly associated with fruit abscission in *C. oleifera* (**Figures 1C, D**). Our previous study also showed that ACC content and ACC activity were significantly increased in the abscission zones (AZs) of abnormal fruits (AF) (Hu et al., 2021). Moreover, 11 key DEGs related to ethylene were identified in this study. The *ACO* and *ACS* were found significantly upregulated in the FAZs of M2 compared with non-abscised FAZs (**Figure 4A**). In addition, we found that *ETR*, *EIN2*, and *ERF* were also upregulated in the ethylene signaling pathway after ethephon treatment (**Figure 4A**). Our results indicate that both the biosynthesis and signal transduction of ethylene are closely related to fruit abscission in *C. oleifera*.

Auxin is also an important hormone in negatively regulating organ abscission (Meir et al., 2015; Kacprzyk et al., 2022), which might be involved in the antagonistic effects of ethylene (Meir et al., 2015). Our results showed that the IAA content of FAZs in M2 was significantly lower than CK2 (**Figure 1B**). Besides, the early auxin response genes *GH3.6* and *SAUR72* were down-regulated after ethephon treatment. These suggest ethephon could decrease the related auxin inhibition of abscission and eventually lead to fruit abscission.

Both TZR and JA have been identified to positively regulate fruit abscission in other plants (Osugi et al., 2017; Gundesli et al., 2020; Fidelibus et al., 2022). Our results showed that the TZR content in FAZs of abscising fruits was significantly upregulated compared with those in non-abscised fruits (**Figure 1B**), which is positively related to ethephon. Transcriptome data also showed the expression of TZR-related genes was up-regulated both in ETH1, ETH2, and M2 (**Figure 4B**). These suggest that TZR might play the synergistic role with ethylene in the abscission of *C. oleifera* fruits. It is interesting that our results showed the JA content significantly decreased in FAZs of M2 when compared with non-abscised fruits (**Figure 1B**), and the expression of JA-related genes was down-regulated (**Figure 4B**). This is contradictory with the effects of JA in grapes (Fidelibus et al., 2022) and needs to be confirmed in future.

### 4.2 Identification of genes involved in cell wall catabolism affecting fruit abscission

The abscission of plant organs occurs at the junction between the to be abscised organ and plant body, that is, the AZs. The changes in AZ cell structure were directly related to the berry abscission of table grapes (Wu et al., 2020). The destruction of the middle lamella is related to the expression of various pectinase enzymes, including polygalacturonases (PGs), pectinate lyases (PLs), and pectin methylesterases (PMEs). These enzymes are believed to open the pectin matrix by reducing the esterification and cross-linking of pectin polymers, allowing other degrading enzymes to enter, leading to cell wall degradation (Kim et al., 2019). In this study, we identified several *PGs* and *PMEs* genes, and the expression levels of these genes were significantly upregulated in soon-to-abscised fruits compared with non-abscised fruits. Studies have shown that exogenous ethephon treatment significantly increased the expression of the *PG* gene in the AZs of citrus and litchi fruits (Peng et al., 2013; Cheng et al., 2015). Interestingly, the *PG2* gene was significantly up-regulated compared with CK2 in both exogenous applications of ethephon and natural abscission in this study. Our result suggests that the *PG2* gene is the key polygalacturonase gene expressed and associated with the degradation of the cell wall of *C. oleifera*.

Cellulase, cellobiohydrolase, and endoglucanase are involved in cell wall modification (Tranbarger et al., 2017). We identified several related genes encoding cellulase, including *CEL* and *CBH*. It is worth noting that during the fruit abscission process of lychee, citrus, and avocado, the *CEL* genes were significantly upregulated (Merelo et al., 2017; Li et al., 2019). In this study, we also found that the *CEL6C* gene encoding exoglucanase was significantly upregulated in M2. In addition, the expression of the *XTH* gene in the FAZs of ETH2 was significantly upregulated with respect to CK2 in this study. Therefore, it is speculated that this gene may be a key cellulase gene in *C. oleifera* fruit abscission.

### 4.3 Regulation of transcription factors during *C. oleifera* fruit abscission

Plant organ abscission is often regulated by multiple genes, in which transcription factors play an important role (Kim et al., 2019). We found that the transcription factors most related to *C. oleifera* fruit abscission belong to the following six gene families: *NAC*, *ERF*, *BZIP*, *BHLH*, *WRKY*, and *MYB*. Among them, *NAC* transcription factors constitute a large plant-specific family that participates in many regulatory and developmental processes, as well as stress responses in several plants, playing a crucial role in regulating development and responding to abiotic and biotic stresses (Hu et al., 2006; Nakashima et al., 2007). Besides, a large number of studies have confirmed that *ERF* transcription factors play an important role in plant organ abscission (Gao et al., 2019, 2020). We found that most of the *NAC* and *ERF* genes were highly expressed in ETH2 or M2 during the fruit abscission of *C. oleifera*, especially the expression of *NAC100* and *ERF114*, which were significantly up-regulated under the conditions of ethephon spraying and natural fruit abscission. Moreover, the WGCNA result showed that there were 5 hub genes related to fruit abscission, among which the transcription factors *NAC100* (unigene0103564) and *ERF114* (unigene0048620) had the strongest gene connectivity, indicating that *NAC100* and *ERF114* had the strongest correlation with fruit abscission. Silencing of transcription factor *SlERF52* can delay the fruit abscission in tomato (Nakano et al., 2014). Transcriptome analysis found the *AcERF1* and *AcNAC48* were significantly involved in fruitlet abscission of *Areca catechu* (Li et al., 2023b). These suggest that *NAC100* and *ERF114* might play important roles in the fruit abscission of *C. oleifera*.

In addition to the above two transcription factors, the WGCNA results showed that transcription factors *BZIP53* (unigene0045875) and *BHLH162* (unigene0147953) were identified as key regulators of fruit abscission in this study. The *BZIP* transcription factor LcHB2 regulates the fruit abscission in litchi (Li et al., 2019). Our results also identified several transcription factors belonging to the BZIP family, such as BZIP*53* and AFT1, which are highly representative of FAZs. According to the heat map of transcription factors, *BZIP53* (unigene0045875) is highly expressed at ETH2 and M2, which is consistent with the results of WGCNA. In addition, we observed that after exogenous ethylene treatment, the expression of the transcription factor *BHLHs* was significantly upregulated compared with that of CK, suggesting that members of the *BHLH* gene family may mediate fruit abscission. Studies have shown that *BHLH18* is involved in regulating low-temperature induced leaf abscission in cassava (Liao et al., 2022), and the abscission of camellia flowers belonging to the same genus as *C. oleifera* is also related to the transcription family *BHLH* (Cai et al., 2022), which further supports our hypothesis. In summary, various transcription factors may have a cascade or synergistic regulatory effect during the fruit abscission process of *C. oleifera*, and the specific mechanisms require further research.

## Data Availability

The datasets presented in this study can be found in online repositories. This data can be found here: https://www.ncbi.nlm.nih.gov/bioproject/PRJNA1101220.
