# Supplementary material for: Transcriptome analysis unravels key pathways and hub genes related to immature fruit abscission in Camellia oleifera
Source: Front Plant Sci. 2024 Aug 9;15:1418358. doi: 10.3389/fpls.2024.1418358 (PMC11341453; doi:10.3389/fpls.2024.1418358)
Supplement: Supplementary file 1 [file DataSheet_1.docx]

**Figure S1** GO functional annotation analysis of CK1 vs ETH1, CK2 vs ETH2, CK2 vs M2, and ETH2 vs M2 differentially expressed gene (DEGs) sets. (A) GO function annotation analysis of DEGs in the CK1 vs ETH1 group; (B) GO function annotation analysis of DEGs in the CK2 vs ETH2 group; (C) GO function annotation analysis of DEGs in the CK2 vs M2 group; (D) GO function annotation analysis of DEGs in the ETH2 vs M2 group.

**Figure S2** KEGG functional annotation analysis of the CK1 vs ETH1, CK2 vs ETH2, CK2 vs M2, and ETH2 vs M2 DEGs sets. (A) KEGG function annotation analysis of DEGs in the CK1 vs ETH1 group; (B) KEGG function annotation analysis of DEGs in the CK2 vs ETH2 group; (C) KEGG function annotation analysis of DEGs in the CK2 vs M2 group; (D) KEGG function annotation analysis of DEGs in the ETH2 vs M2 group.

**Figure S3** WGCNA analysis of KEGG enrichment analysis of fruit abscission related modules. (A) KEGG enrichment analysis of antiquewhite4 module; (B) KEGG enrichment analysis of coral2 module; (C) KEGG enrichment analysis of lightcyan1 module.

**Figure S4** qRT-PCR analysis of key TFs (*CoERF114* and *CoNAC100*) in fruit abscission. Small letters (a–c) are shown for means with significant differences (*P* < 0.05).
